# Forward Computational Modeling of Respiratory Airflow

**DOI:** 10.3390/app142411591

**Published:** 2024-12-12

**Authors:** Emmanuel A. Akor, Bing Han, Mingchao Cai, Ching-Long Lin, David W. Kaczka

**Affiliations:** 1 Roy J. Carver Department of Biomedical Engineering, University of Iowa, Iowa City, IA 52242, USA;; 2 Department of Mathematics, Morgan State University, Baltimore, MD 21251, USA;; 3 Department of Anesthesia, University of Iowa, Iowa City, IA 52242, USA; 4 Department of Mechanical Engineering, University of Iowa, Iowa City, IA 52242, USA; 5 Department of Radiology, University of Iowa, Iowa City, IA 52242, USA

**Keywords:** airflow, computational fluid dynamics, airway tree, computed tomography, fluid structure interaction

## Abstract

The simulation of gas flow in the bronchial tree using computational fluid dynamics (CFD) has become a useful tool for the analysis of gas flow mechanics, structural deformation, ventilation, and particle deposition for drug delivery during spontaneous and assisted breathing. CFD allows for new hypotheses to be tested *in silico*, and detailed results generated without performing expensive experimental procedures that could be potentially harmful to patients. Such computational techniques are also useful for analyzing structure–function relationships in healthy and diseased lungs, assessing regional ventilation at various time points over the course of clinical treatment, or elucidating the changes in airflow patterns over the life span. CFD has also allowed for the development and use of image-based (i.e., patient-specific) models of three-dimensional (3D) airway trees with realistic boundary conditions to achieve more meaningful and personalized data that may be useful for planning effective treatment protocols. This focused review will present a summary of the techniques used in generating realistic 3D airway tree models, the limitations of such models, and the methodologies used for CFD airflow simulation. We will discuss mathematical and image-based geometric models, as well as the various boundary conditions that may be imposed on these geometric models. The results from simulations utilizing mathematical and image-based geometric models of the airway tree will also be discussed in terms of similarities to actual gas flow in the human lung.

## 1. Introduction

Computational fluid dynamics (CFD) refers to computer-based modeling tools for the simulation and study of fluids in motion. CFD employs numerical methods and algorithms to solve and analyze the governing equations of fluid flow [1]. For simulating gas flows in the mammalian airway tree, CFD is useful for evaluating the impact of various structural derangements on pulmonary function, as well as predicting the regional distributions of airflow, airway wall shear stress, and particle deposition under healthy and diseased conditions [2–4]. CFD simulations allow for clinical hypotheses to be evaluated in silico, which would otherwise be impracticable, potentially harmful, or cost-prohibitive for in vivo or ex vivo experimentation. CFD also provides high levels of anatomic detail for analyzing flows in complex branching systems, such as vascular and airway trees, or subunits of those systems that would be impossible to access through direct measurement [1]. To replicate actual physiologic behavior, such simulations must utilize valid three-dimensional (3D) geometric structures, as well as realistically functional boundary conditions. The complexity of the mammalian airway tree places several constraints on CFD simulations, such as imaging resolution, the imposition of physiologic boundary conditions, turbulence in the upper airways, and the limitations of the existing computational resources [5].

In 1963, Weibel conceptualized the human airway tree with 23 dichotomously branching airway generations comprised of conducting and respiratory zones [6]. The conducting zones (trachea to terminal bronchioles) comprised the first 16 generations, while the respiratory zone comprises 7 generations. Both zones make up more than 65,536 distinct airway segments in the human lung, which become progressively smaller in dimension further from the trachea. Advection and diffusion serve as the two primary modes of gas transport in the lungs, and thus are incorporated into CFD simulations of respiratory air flow. The dominant mode of transport in the conducting zone is advection (i.e., bulk flow), for which there is minimal-to-no gas exchange. However in the respiratory zone, the dominant mode of transport is diffusion, *via* the movement of oxygen (O_2_) and carbon dioxide (CO_2_) down their concentration gradients across the blood-gas barrier [1]. In the adult human, the volume of the conducting zone averages about 150 mL, while the respiratory zone has a volume of about 2.5 to 3 L at functional residual capacity [7]. During spontaneous breathing, the volume change of the respiratory zone is significant, while that of the conducting zone is minimal, whereas in assisted breathing, both volumes change considerably, especially when high positive pressures are utilized [8]. The goal of CFD for simulation of respiratory phenomena is to mimic the behavior of gas flow (in the conducting airway and respiratory zones) and/or pulmonary blood flow during inspiratory and expiratory cycles, whether such breathing is spontaneous or assisted. During spontaneous breathing, inspiration is initiated by the contraction of the diaphragm and external intercostal muscles, resulting in the expansion of the thoracic cavity. This expansion results in a decrease in alveolar pressure, and an increase in the pressure gradient between the distal lung and atmosphere, accordingly, air flows into the lungs *via* advection. Under normal breathing conditions, expiration results from the passive elastic recoil of the lung tissues and chest wall. This recoil decreases the volume of the thoracic cavity, leading to an increase in alveolar pressure relative to the atmosphere, and establishes a pressure gradient to move air out of the lung. These inspiratory and expiratory phases induce non-uniform volume changes throughout the lung tissues, because of the inherent heterogeneity in regional mechanical properties [9]. This distribution of airflow in the lungs (during spontaneous breathing) initiated by the pressure gradient is influenced by both resistance of the airway pathways and the compliance of the lung tissue.

Several studies have relied on the symmetric [6] and asymmetric [10–12] airway trees to understand the distributions of airflow, ventilation, and other mechanical properties in the lungs, given their simplicity in structure, since both models assume regular dichotomous branching. The aforementioned Weibel model assumes symmetry in branch dimensions (i.e., length and diameter) and bifurcation angles of parent-daughter segments in a particular generation [6]. The generation number for each branch in the tree is assigned relative to its parent branch, with the trachea assigned as Generation 0. Each subsequent daughter branch is assigned a generation higher than the previous maternal branch.

By contrast, the models of Horsfield and co-workers [12, 13] assume asymmetry in bifurcation and branch dimension, based on a recursion index Δ. The trachea is assigned the highest order number, with one daughter branch an order lower and the other determined by an assigned value for Δ, representing the difference in order numbers for daughter branches of the same generation. For computational modeling, tracheobronchial trees generated using either symmetric or asymmetric designs are often modeled as a 1D flow field (i.e., axial), integrating governing equations for pressure-flow relationships over the cross-sectional area of the airway branches [10, 14].

Most airflow simulations of the lung focus on small subsections of the airway tree and/or a fixed mesh for tissue behavior [2, 15–18]. Simulations using such models usually rely on the assumption of smooth cylindrical tubes for the airway branches [10] whereas the human airway cross-section is quite irregular in shape [17]. Moreover, the airway airflow simulations are often simplified, and do not accurately reflect the flow mechanics associated with heterogenous and irregular airway branching. Neither the Weibel nor Horsfield models account for the three-dimensional (3D) spatial arrangement of the airways. Furthermore, the design of both models is based on airway tree casts [12, 19], which may introduce geometric distortion to the parent-daughter branch angles and tissue structures.

Given recent advancements in subject-specific *in silico* simulations for potential clinical decision-making [1], there is a need for more structurally and spatially realistic 3D models for predicting response to therapy or treatment outcomes, since the branching structural pattern of the airway tree influences the distribution of airflow. Not only will more advanced 3D geometric models be a significant improvement to understanding the mechanics of airflow in the lung, but they will also have utility for enabling high-fidelity simulations of airflow in diseased lungs, compared to the more conceptually idealized Weibel and Horsfield structures [10].

In this focused review of respiratory airflow modeling, we will summarize the techniques and limitations used for generating realistic, 3D geometric models of the mammalian lung, as well as various techniques used for computational airflow simulations based on mathematical and image-based geometric models, as well as the boundary conditions imposed. Fluid-structure interactions, and the nature of airflow (i.e., laminar, transitional, and turbulent), at different regions of the lung will be considered, as well as the impact of various assumed flow paradigms on ventilation distribution and other functional indices. Finally, we will discuss the relationship between ventilation and perfusion, validation techniques used to assess the accuracy of the current CFD methodologies, and considerations for improving the accuracy of respiratory CFD simulations.

## 2. Mathematical Structural and Spatial Geometric Models

Prior to advancements in medical imaging technologies with high spatial resolution, mathematically based geometric models of the lungs were used to develop airway trees for CFD simulations. Based on initial designs of Weibel [6] and Horsfield et al. [12, 20], geometric models have been created to account for asymmetric bifurcation angles, cross-sectional areas, diameter, and length of airway branches. One such model by Kitaoka et al. [15] is based on two simple principles that determine airway branch dimension and spatial dimensional arrangements for a dichotomous branching airway tree. This model assumes that the amount of fluid delivery through a branch is proportional to the volume of the region it supplies and that the terminal branches of the airway tree are homogeneously arranged within the organ [15].

The relationship between flow rate and airway branch diameter is established based on power law, assuming circular airway segments with constant diameters [15]. Both parameters are normalized by their respective values in the parent branch (Equation 1).

(1) $${\dot{V}}^{\prime }={\left(d^{\prime }\right)}^{n}$$

where ${\dot{V}}^{\prime }$ and $d^{\prime }$ are the normalized flowrate and diameters, respectively, for any daughter branch, and n is the diameter exponent. Based on the principle of minimum energy loss for steady laminar flow in a rigid, circular conduit, n is empirically assigned a value of 3 [21]. According to Murray’s law [22], the diameter relationship between the parent and daughter branches is given as

(2) $${d_{0}}^{n}={d_{1}}^{n}+{d_{2}}^{n}+\ldots \ldots +{d_{i}}^{n}$$

where $d_{0},\;d_{1},\;d_{2}$, and $d_{i}$ are the diameters of the parent, first, second, and *i*^th^ daughter branch respectively. Furthermore, a ratio f can be defined as the flow fraction (between the daughter and parent branch) in the first daughter branch, and the diameters of the daughter branches are directly related to the parent branch as

(3) d1=d0f1n

(4) d2=d01-f1n

Hence, if the dimensions of both daughter branches are unknown, and a desired flow is assumed in either branch, the dimensions of both branches can be obtained. Murray [22] further derived the relationship between the diameter d of the parent and daughter branches and the bifurcation angles as

(5) $$\text{cos}\;\theta_{1}=\frac{{d_{0}}^{4}+{d_{1}}^{4}-{d_{2}}^{4}}{2{d_{0}}^{2}{d_{1}}^{2}}$$

(6) $$\text{cos}\;\theta_{2}=\frac{{d_{0}}^{4}+{d_{2}}^{4}-{d_{1}}^{4}}{2{d_{0}}^{2}{d_{2}}^{2}}$$

where $\theta_{1}$ and $\theta_{2}$ are the bifurcation angles between parent branch and the first and second daughter branches respectively. When the flow fraction f for one of the daughter branches is known, the equations for the branching angles become:

(7) cosθ1=1+f4n-1-f4n2f2n

(8) cosθ2=1+1-f4n-f4n2(1-f)2n

**Figure 1.**
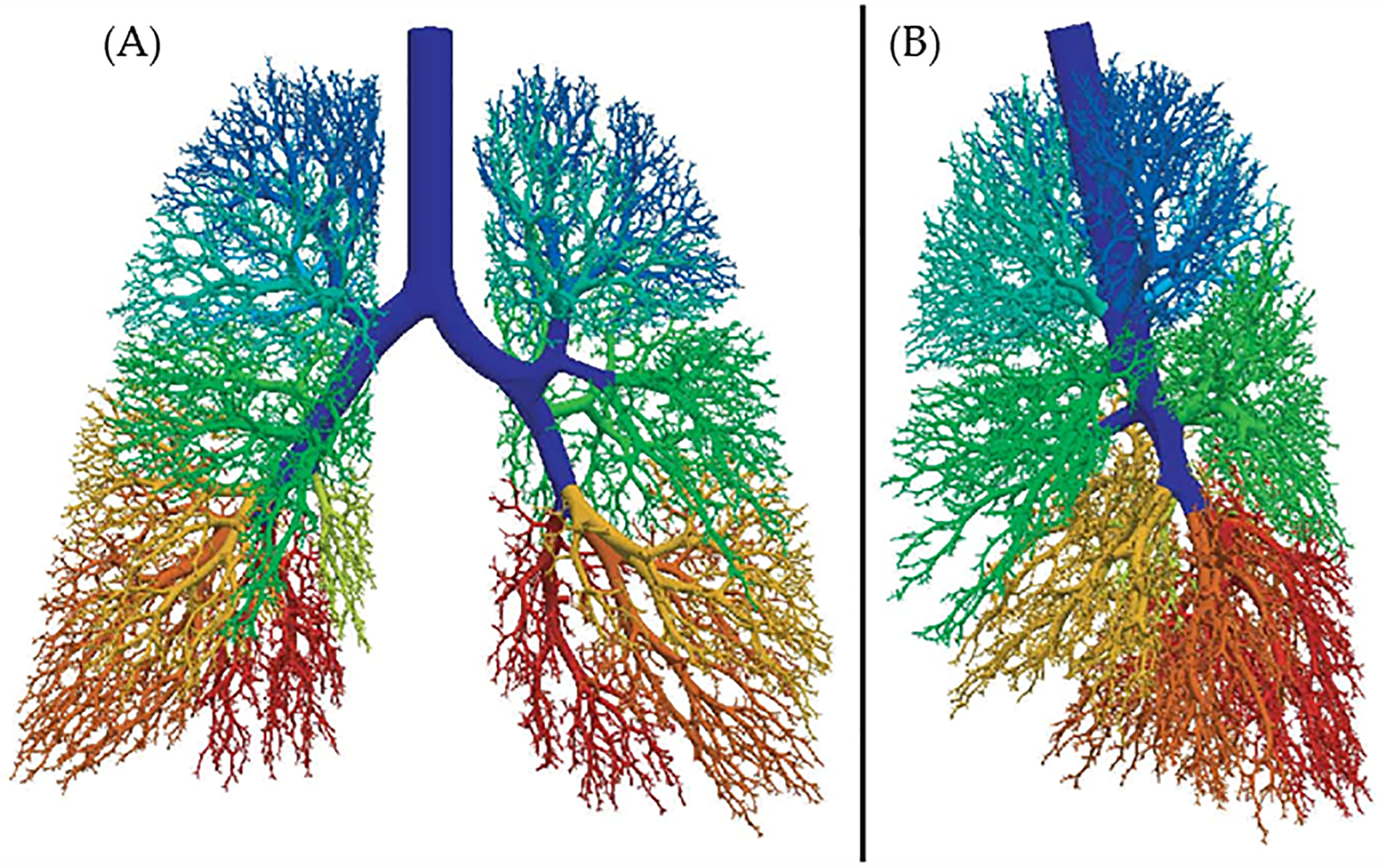
Projected images showing the three dimensional structural and spatial model of the airway tree based on mathematical algorithm developed by Kitaoka et al [27]. There are about 30,000 terminal branches and 54,611 total branches in this model. The airway branches distal to given segmental bronchi are shown by the same color. (A) anterior view (B) lateral view. This work is made available under the terms of the Creative Commons Attribution CC BY 4.0 (https://creativecommons.org/licenses/by/4.0/).

The algorithm for generating this geometric model is governed by basic and supplementary rules [21] which were used to successfully generate an airway tree (Figure 1) with approximately 54,000 branches utilizing the above equations.

Tena et al. [23] generated a 3D geometric model of the lungs based on these principles, for an airway tree having 65,536 branches, and developed a CFD model of orally inhaled medication that could predict drug deposition characteristics and physiological parameters in the lungs, showing the utility of this geometric model.

**Figure 2.**
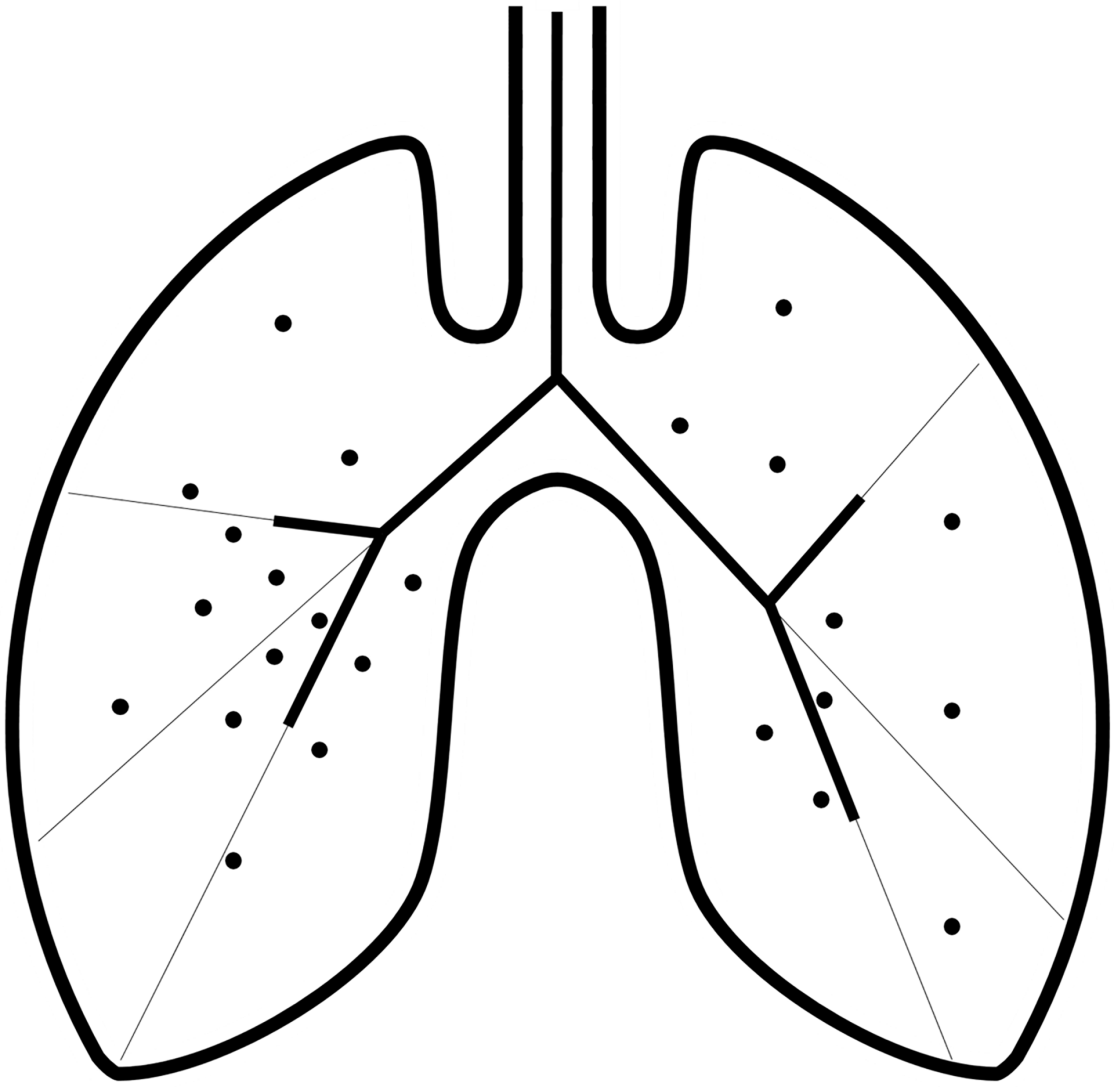
A two-dimensional branching network of the lung airway tree showing the Monte Carlo method for any distributive system. The dots represent random points in 2D space that determine how the network branches. The thick lines within the enclosed lung surface represent the airway branches for the first two generations, and the thin lines are the dividing lines that are generated from the calculation of the center of mass of the points in any given region.

The airway tree is often assumed to be fractal in nature (i.e., having detailed self-similar structures at different scales), with a large degree of heterogeneity and lack of a characteristic scale. Mandelbrot [24] theorized the nature of the airway tree as a fractal-based bifurcating tree with a rectangular shape with a dimension of $\sqrt{2}\times 1$ [25, 26]. Although Mandelbrot’s model provides useful insights into the bifurcation of any distributive system, it is limited in its use to the $\sqrt{2}\times 1$ rectangular geometry, and thus cannot be directly applied to the lung [26]. As a result of this deficiency, Nelson and Manchester [25] developed an area-splitting algorithm for dichotomous branching, which splits any given area in half and assigns the branch length to be half of the effective diameter of the area. This iterative algorithm terminates when a rectangular area with a ratio of $\sqrt{2}\times 1$ is attained, and then Mandelbrot’s algorithm is implemented. This method is computationally expensive, given the computation of $2^{23}-1$ distinct irregular areas if Weibel’s morphometry is assumed. Hence, Wang et al. [26] described a Monte Carlo method for any distributive system (Figure 2), with the conceptual criterion that any location in this system must be reachable through a pathway.

This method is computationally efficient and utilizes random points inside a defined two-dimensional (2D) area to generate branches that serve those points based on four principles:

1. The center of mass of the points is found by calculating the mean of the coordinate positions of all the points in any given area.
2. A dividing line that begins from the starting point of the branching system, goes through the center of mass, and ends at the boundary of the given area is drawn. A branch is then drawn from the starting point along the diving line with a predefined length.
3. This dividing line separates the initial area into two areas containing points.
4. The process is iterative until a computed area contains only one point.

This Monte Carlo method requires that the number of generated airway branches is dependent on the number of initial spatial points. However, knowing the number of points for any distributive system is ambiguous. Moreover, the bifurcation angles of the branches cannot be specified, since they follow from the dividing line at each iteration. Accordingly, some of the angles generated will be unrealistic, and not representative of an actual mammalian airway tree.

Tawhai et al. [28] expanded on the Monte Carlo method, to include a 3D spatial airway distributive system with some modifications. A dividing plane is used in 3D space, to connect the starting point to the end of the boundary through the center of mass, as opposed to a 2D line. A surface mesh derived from a 3D computed tomography (CT) or magnetic resonance imaging (MRI) scan is utilized in defining the host volume. To make the airway tree branching more realistic, the volume is divided into five subregions representing the human lobe volumes. The pathway from the trachea to the lobar bronchus for each of the five subregions is based on the model of Horsfield et al. [20], but modified to include 3D spatial branching. The ends of the lobar bronchi are used as the starting point for the distributive system, ensuring that additional lobar bronchi are not generated. However, the choice for the dividing plane is infinite, since any plane in space can connect two distinct points. Hence the vector in the direction of the parent branch and the resultant vector of the coordinate of the center of mass determines the dividing plane. Tawhai et al. [28] further modified the model to rely completely on imaging data.

While the aforementioned 3D geometric models represent improvements over those of Weibel and Horsfield et al., they are devoid of subject-specificity for predicting clinically relevant structure-function interactions that may have diagnostic or treatment monitoring utility. Because airway geometry and gas flow distribution are highly correlated, intrasubject variation in airway geometry is essential for potential diagnoses and clinical management, making patient-specific geometric models based on imaging useful for the analysis of pulmonary airflow, particle tracking, and regional ventilation [5, 10, 16]. Nonetheless, these models provide a generalized understanding of airflow in a distributive branching system, from which realistic behavior of the respiratory system might be inferred.

## 3. Image-based Structural and Spatial Geometric Models

Medical imaging has provided the opportunity to visualize and track lung pathologies and phenotypical changes over time, response to treatment protocols, and allow for better insight into the functional behavior of the lung [29–32]. Three-dimensional anatomic models of the lungs can now be generated rapidly based on a single breath hold image [33]. Both X-ray computed tomography (CT) and magnetic resonance imaging (MRI) have allowed for the creation of high-resolution, subject-specific 3D structural, spatial, and functional models of airflow in the lung. These subject-specific models are useful for visualizing and studying personalized changes in airflow pattern, ventilation, particle deposition, and lung tissue deformation [4, 29, 34–36]. Such models also have utility in precision medicine, for testing new hypotheses and guiding clinical management. Moreover, techniques that quantify regional changes at distinct locations can be used to assess associations with measures of global lung function, such as plethysmography or spirometry. CT and MRI allow for the coupling of both CFD and ventilation distribution [14], as well as the matching of perfusion and ventilation distributions [1, 37]. Nowak et al. compared functional simulations from a modified 3D Weibel model that included spatial branching, and an image-based model from a CT scan of a cadaver lung cast, both having four branching generations [38]. They also demonstrated that the velocity fields in the image-based model are more complicated (i.e. more heterogeneously distributed) than in the Weibel model for airway segments in the same generation. The results further showed significant differences in regions of particle deposition between the two models. Although both models showed particle deposition at airway bifurcations, the image-based model showed particle deposition on airway walls while the Weibel model showed little to no particle wall deposition. This was attributed to the absence of airway curvature and surface irregularities in the Weibel model, which makes it insufficient to predict particle deposition.

Although CT imaging can yield high spatial and temporal image resolutions, high radiation doses are required to evaluate various structural pathologies of the lung, thus limiting the availability of images from which 3D subject-specific models can be generated [39]. While MRI has advantages over CT regarding radiation exposure, it traditionally has a limited ability to depict lung anatomical details. Hence, new methods to optimize CT imaging with reduced radiation exposure while simultaneously acquiring high-resolution imaging scans are necessary. Both CT and MRI can be used to incorporate structural and functional changes associated with lung disease and pathology.

### 3.1. Lung Segmentation

To utilize medical images in generating 3D geometric models that are useful for CFD simulation, whole lung and airway tree segmentations from the chest wall and mediastinum must first be performed. The goal of lung segmentation is to separate voxels corresponding to the lung from those of other anatomic structures [33]. Such image segmentation can be achieved manually or automatically and serves as an antecedent for quantitative image analysis or computational modeling. The steep intensity gradient (i.e., low-intensity value of the lung and high-intensity surrounding chest wall) makes it relatively simple to segment lung tissues for a healthy subject, using automated thresholding techniques [40]. However, the situation becomes far more complicated for an injured or inflamed lung, in which high-intensity voxel values of consolidated and derecruited lung tissue are almost indistinguishable from the surrounding chest wall and mediastinal contents. Automated segmentation is generally preferred over manual segmentation, since the latter is tedious, time-consuming, and impractical for dynamic imaging. Moreover, manual segmentations may include significant interobserver variability, especially when analyzing large datasets [33, 41].

Hu et al. [33] developed an algorithm for automated whole-lung segmentation for healthy subjects based on three main steps. First, optimal gray-level thresholding is applied to the image scan, then the anterior and posterior junctions of the lungs are identified to distinguish the left and right lung, and finally, the boundary of the lung is smoothed. The optimal gray-level threshold segments the 3D image into body and non-body voxels by an iterative method. An initial threshold value *T*_0_ is assigned for segmentation, and the next threshold value $T_{i}$ is calculated by taking the average of the mean intensities in the body ($I_{b}$) and non-body ($I_{n}$) voxels. The process continues until the threshold value,

(9) $$T_{i}=T_{i-1}$$

where

(10) $$T_{i}=\frac{\left(I_{b,i}+I_{n,i}\right)}{2}$$

Using this method, low-intensity voxels within the lungs or other structures (i.e., bowel gas) can be successfully segmented. The lung is identified using connectivity and topologic analyses, that retain only volumes greater than a predetermined minimum volume. This optimal threshold is unique to the specific dataset, from which the whole lung can be segmented. This is important since it considers the existence of intersubject variability in tissue intensities. The left and right lungs are separated using a dynamic programming technique that locates the anterior and posterior junction lines for each transverse slice and the boundary for each lung is smoothed [33, 42].

Gerard et al. [41] developed a convolutional neural network (CNN) for whole lung segmentation in various mammalian species with both healthy and diseased conditions. Their technique was based on deep learning, allowing the CNN to extract certain features from spatially correlated data and to make predictions of anatomic boundaries. For whole lung segmentation, the network was trained with manually segmented image datasets from humans, which was then extended to porcine, canine, and ovine subjects using transfer learning. The manually segmented image datasets (i.e., ground truth) were generated by first using gray-level thresholding, then corrected by expert image analysts.

### 3.2. Lobar Segmentation

Human lungs have five distinct lobes. The right lung has upper, middle, and lower lobes, while the left lung has only upper and lower lobes. Segmentation of distinct lobes may be performed to aid airway branch identification during automatic airway segmentation, and to quantify lobar structural and functional changes during breathing. Such segmentation is accomplished by identifying the physical boundaries (i.e., fissures) that separate the five lobes into different compartments. The right upper and middle lobes, the right middle and lower lobes, and the left upper and lower lobes are separated by the right horizontal fissure, the right oblique fissure, and the left oblique fissure, respectively.

### 3.3. Airway Segmentation

After lobar segmentation, the airway tree is automatically segmented out of the whole lung structure, else manual or semi-automatic airway tree segmentation is required. A given segmented airway tree is converted into a 3D geometric structure for CFD simulations by conforming the model geometry to the surface data of the airways and/or creating a 1D centerline airway tree in 3D space [5]. To utilize 1D centerline airway for CFD Simulation, diameters must be defined throughout the entire structure based on image measurements of the airway in a plane orthogonal to the central axis [5]. Such 3D geometric structures can be utilized directly if saved in a format recognized by CFD simulation software (e.g., Ansys, SOLIDWORKS, COMSOL Multiphysics, among others).

**Figure 3.**
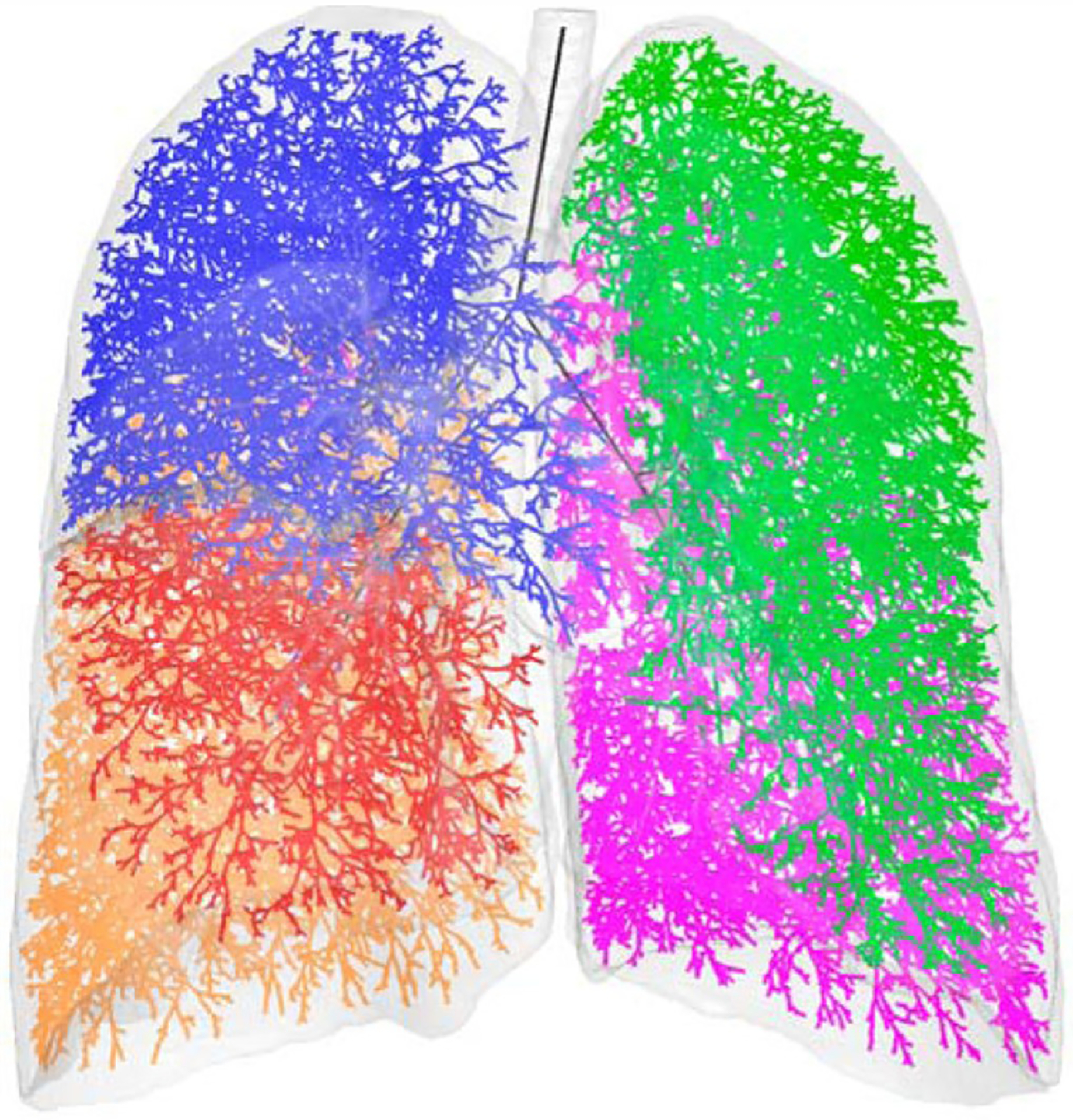
3D Spatial, 1D Structural Model generated using the Volume Filling Method. The generated airways are shown in different colors for each lobe: green for left upper lobe (LUL), magenta for left lower lobe (LLL), blue for right upper lobe (RUL), red for right middle lobe (RML) and orange for right lower lobe (RLL). Modified from reference [44], with permission.

The geometric accuracy of airway tree models generated from 3D medical images is dependent on spatial resolution. Hence, segmentations are only feasible for about four to five generations. To overcome this limitation, Tawhai et al. [43] developed an image-based volume-filling branching (VFB) method to create an entire airway tree model with high anatomic fidelity. This method was based on the modification of the Monte Carlo technique for a distributive system as discussed above. The generated geometric model accounts for the asymmetry between parent and daughter airway segment lengths, diameters, and branching angles. As opposed to utilizing the methodology of Horsfield and Cumming [20] for the generation of the central airway geometry with ends that served as the initial points for the VFB method [28], the central airway tree is semi-automatically segmented out to the fifth generation from images obtained at total lung capacity (TLC). Lung lobes are segmented as well, serving as host volumes which are filled with uniformly spaced grids of seed points. Each seed represents the location of an acinus in space. Centerline information is extracted from the segmented central airway, and the end of the centerline serves as the initial point while the surface of the host volume (lobar surface) serves as the boundary. The seed containing volumes are split recursively into subregions as described earlier, and the seed points are associated with the closest terminal branch. This iterative process repeats until the length of a branch is less than a predefined terminal length or only one seed point left in the subregion, leaving room for alveoli. The VFB hence generates a 1D-structural, 3D-spatial geometry for the whole lung (Figure 3).

Lin and co-workers [16, 45, 46] used this VFB methodology to develop a multiscale image-based model for the whole lung. It is multiscale because it incorporates both 3D and 1D geometric structures in a single model. After the generation of the entire 1D structural airway tree using the VFB method, any airway branch pathway from the trachea to a terminal bronchiole can be selected and converted to a 3D structure. The 3D mesh is only created for the pathway, while the rest of the tree can be described as a 1D structure. This 3D-1D structural combination allows for air flow simulations and analyses in specific regions of the lung that cannot be obtained by 1D simulations alone.

## 4. Boundary Conditions and Simulation Methods

The CFD simulations of gas flow in the airway tree are usually calculated numerically based on the Navier–Stokes equation using a CFD solver. Given the regional variations in oxygen, carbon dioxide, and nitrogen within the lung and over the breathing cycle, the actual density and viscosity of gases may not be uniform throughout the organ. Air density and viscosity are also temperature-dependent. However, for the simplicity of the simulations, air is typically assumed to be incompressible and Newtonian, with a nominal density ρ of 1.225 kg m^−3^ and a viscosity μ of 1.81×10–5 kg m^−1^ s^−1^.

Mesh-free methods can also be used for respiratory flow simulations, such as smoothed particle hydrodynamics (SPH) [47, 48], element-free Galerkin method (EFG) [49, 50], and the moving particle semi-implicit method (MPS) [51]. Discretizing the flow region remains a commonly used approach to solving the Navier–Stokes equation. Examples of these discretization methods include the Finite Element Method (FEM), Finite Volume Method (FVM), and Finite Difference Method (FDM) [52]. Generally, the finer the mesh, the more spatially detailed and accurate the solution becomes. However, there are a few exceptions where the resulting solution is unchanged [53]. This increase in detail and accuracy is, nonetheless, accompanied by an increase in computational time and resources. The numerical solution is solved iteratively until convergence is attained (i.e., when the residual is less than a pre-assigned value, typically on the order of 10–5).

Because of the anatomic complexity of the upper airway (i.e., nasal and oral regions), most CFD simulations rely on airway models with an inlet beginning along the trachea [16]. Airflow profiles in the intra- and extrathoracic regions are described using dimensionless numbers, such as the Reynolds (Re) and Womersley (α) numbers.

(11) $$\text{Re}=\frac{\rho \overset{-}{v}d}{\mu }$$

(12) $$\alpha =\frac{d}{2}\sqrt{\frac{\omega \rho }{\mu }}$$

where ρ is the fluid density, d is the diameter of the airway segment, $\overset{-}{v}$ is the average velocity with the segment, and ω is the angular frequency of gas cycling in units of rad s^−1^.

For steady airflow, the regime is considered laminar for Re less than 2000, transitional for Re between 2000 and 3000, and turbulent for Re greater than 3000. For oscillatory airflow, the regime is considered quasi-steady for α less than 1 and unsteady for α greater than 10. Laminar flow is assumed when resistive pressure losses due to air viscosity dominate over inertial pressures. When airflow is turbulent, either Reynolds-averaged Navier-Stokes (RANS), large eddy simulation (LES), or direct numerical simulation (DNS) computational techniques may be employed. RANS (e.g., k-ε, k-ω, SST) filters turbulent eddies using time averaging, LES filters small turbulent eddies using spatial averaging, and DNS directly solves the incompressible Navier-Stokes equations. Either of these three computational techniques can be utilized, depending on the complexity of result needed and the available computational resources. RANS has a low computational cost, is suitable for steady-state simulations, and well-developed for models incorporating turbulent flow. However, it is less accurate for unsteady and complex flows with strong separation or large vortices. LES has better accuracy for unsteady flows, is applicable to complex flow problems, and has more precision than RANS, but comes with a higher computational cost. DNS has the highest accuracy, resolving all flow structures, but has extremely high computational cost, hence it is used for simplified flow problems.

Airflow is considered fully developed or developing based on the extent to which the velocity profile is formed. A fully developed airflow region can be assumed at any point in the airway segment where the length from the entrance (*L*_*e*_) is far greater than the diameter of the segment. More specifically, a fully developed region can be assumed at a length greater than (*L*_*e*_) (Equations 13 and 14), the distance where the shear stress reaches 2% of the fully developed value [54].

(13) $$\frac{L_{e}}{d}=0.05\;\text{Re},\;\text{Re}\;<2000\;(\text{Laminar}\;\text{flow})$$

(14) $$\frac{L_{e}}{d}=50\;\text{Re},\;\text{Re}>2000\;(\text{Turbulent}\;\text{flow})$$

Steady inspiratory flow at the inlet is often assumed using various volume flow rate or pressure values, and uniform pressure or velocity at the outlets for simplicity. The resulting solutions from these simulations, albeit mathematically accurate, are not physiologically representative of the mechanical function of the lungs nor are they subject-specific. Nonetheless, they can be utilized to give insights into the changes in structure and function of healthy vs. injured lung cases. Depending on the aim of the study, there are several computational simplifications that may also be implemented.

For analyzing the relationship of structural and functional alterations in healthy vs. severe asthmatic lungs, Choi et al., [55] utilized the Galerkin finite element method to discretize the airway tree and imposed a steady inspiratory flow rate of 3.27 × 10^−4^ m^3^ s^−1^ (about 20 L min^−1^) and non-uniform flow rates at the outlets. This inspiratory flow rate value is equivalent to the peak flow rate of a sinusoidal waveform with a tidal volume of 0.5 L and a period of 4.8 s. It was concluded that constricted airways in asthmatic patients contribute to higher wall shear stress, elevated pressure drop, and an increase in particle deposition [55]. Although unsteady inspiratory inlet value is ideal, a steady inlet value will suffice for this analysis because the goal is to determine the differences in airflow characteristics as a function of the changes in airway structure. Tena et al. [23] performed a similar simulation by utilizing a User Defined Function (UDF) to simulate airflow in 8 bifurcations of the airway tree obtained from Kitaoka’s modified Horsfield model. To achieve this, the entry boundary conditions (inlet flow rate) were 15, 30, and 75 L min^−1^, while the exit boundary conditions at the airway tree endings were imposed on their truncated homologs and set to a relative pressure of zero. Tena et al. suggested that setting the boundary values of the truncated homologs to relative pressures of zero is unimportant in a bid to understand the relative difference in airflow behavior in different airways [23]. Since the same structure was utilized for all three simulations, the outlet boundary values need to be the same. And since the goal of the study was to understand the impact of changes in inlet flow rate on airflow mechanics, these assumptions suffice.

Another approach for forward computational simulation of the respiratory system entails utilizing results from inverse modeling. Poorbahrami et al. [56] utilized image-based anatomical models of three female human subjects sectioned into subject-specific image-based 3D conducting airways and 1D distal generic airways [56]. The conducting airways were segmented from the 3D CT image scans using SimVascular (an open-source software, https://simvascular.github.io/, accessed on 11/22/2024) and spanned the trachea to the most distal conducting airways visible from the CT scan. However, simulations were only performed for a few generations (8, 11, and 9 for the three models utilized). It was assumed that the boundary conditions at the distal outlets of the conducting airways could not be measured experimentally. Thus, an inverse modeling technique was utilized to get the global resistance and compliance values of the lungs. Using the global resistance and compliance values, the lung periphery was represented by a resistor and capacitor connected in series, driven by a time-dependent pressure value.

While patient-specific 3D models have been utilized in some studies [57–60], the results would not be physiologically accurate because of the heterogeneity in pressure, volume flow rate, resistance, and compliance at different regions of the lungs. Hence, utilizing global or constant values for the outlet parameters will not suffice for a valid analysis of the lung function, even more so for an injured lung. To understand correctly the relative differences in airflow characteristics, relatively varying boundary conditions must be utilized to mimic the inhomogeneity in the lung parenchyma [30, 32, 61]. Also, simulating the entire lung structure using only a few bifurcations might provide approximate pressure and velocity values in the central airway tree, but this method will not help in accurately understanding the distribution of flow in more peripheral regions of the lungs. Furthermore, the assumption of steady inspiratory flow at the inlet of the airway tree is not representative of physiological breathing. As such, oscillatory inspiratory flow and subject-specific outlet boundary conditions may be imposed on the airway tree model, to have a solution that more closely represents lung physiology.

**Figure 4.**
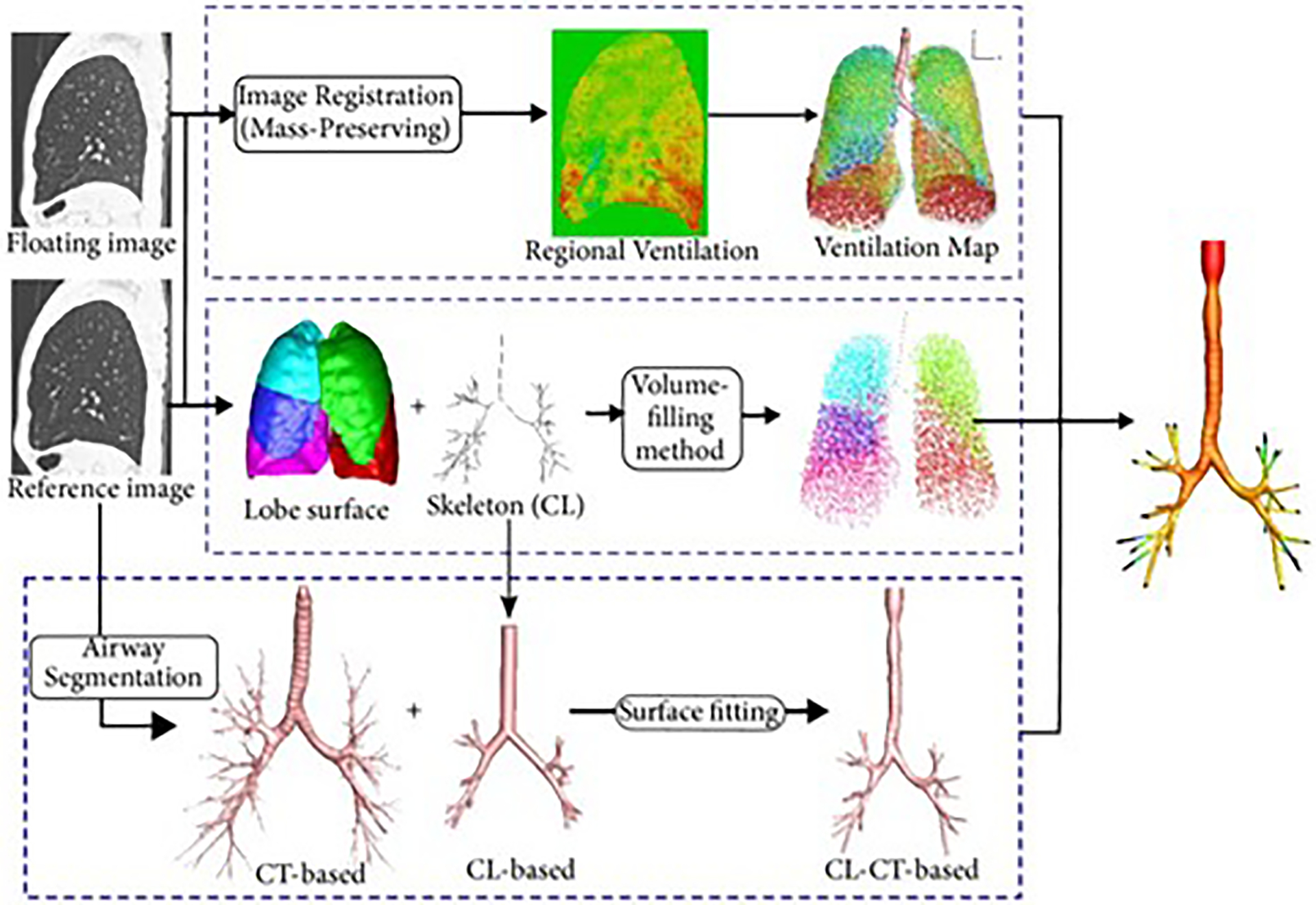
Workflow for attaining subject-specific boundary flow conditions at the distal airway tree [55]. Regional ventilation is obtained from the registration of two 3D images taken at different time points during inhalation, and matched to the structural 1D airway tree using the volume filling branching method. For both regional ventilation and the ventilation map, cool colors correspond to regions of high ventilation, while warm colors correspond to regions of low ventilation. For the volume-filling method, the five different colors correspond to distinct anatomic lobes. This work is made available under the terms of the Creative Commons Attribution CC BY 4.0 (https://creativecommons.org/licenses/by/4.0/).

Yin et al. [2] applied a novel image-based method utilizing subject-specific boundary conditions at the central airway tree endings (Figure 4).

**Figure 5.**
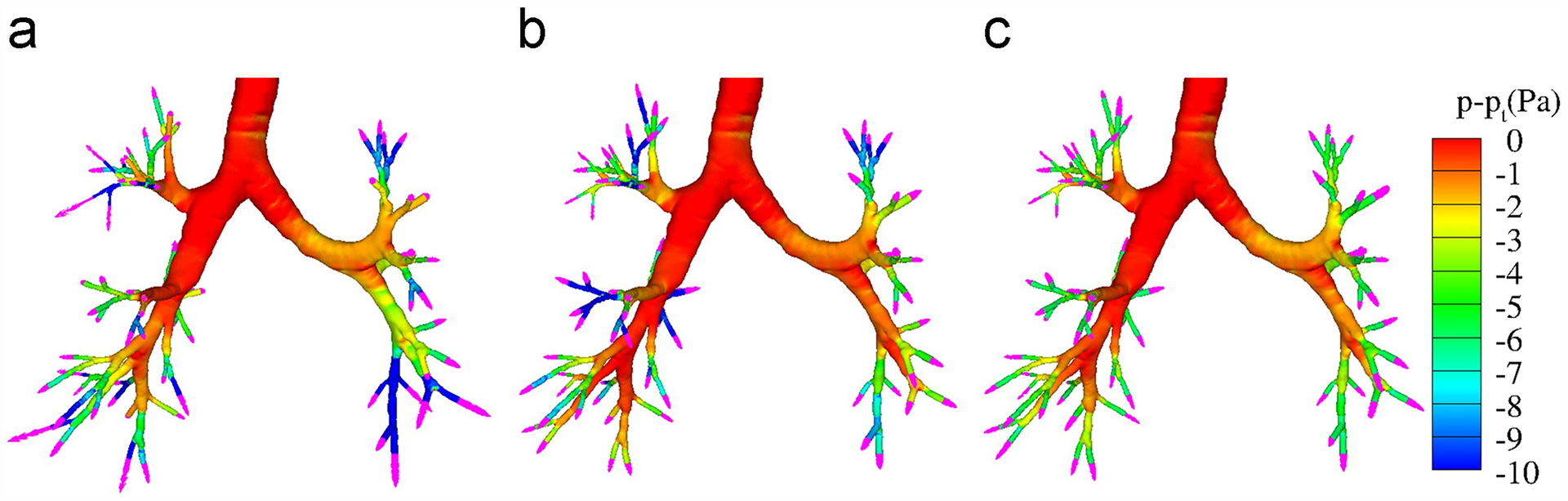
Velocity vectors (pink) and pressure drop distributions in a three-dimensional model with: (a) patient-specific; (b) uniform velocity; and (c) uniform pressure boundary conditions at the distal airway outlets. The patient-specific boundary condition yielded a greater pressure drop in the left lower lobe and the right lower lobe compared with the uniform velocity condition, the uniform velocity boundary condition yielded a greater pressure drop in the right middle lobe than the others, and the uniform pressure boundary condition yielded a uniform pressure drop across all five lobes. From reference [2], with permission.

Two 3D-CT images taken at different time points during inflation were matched using a mass-preserving image registration technique and a map of regional ventilation was obtained. A 1D centerline airway tree was then generated from the distal conducting airway tree segmentation of the larger image using the volume-filling algorithm developed by Tawhai et al. [43] that fills the lung lobes with bifurcating 1D trees. The 1D airway tree is then matched with the regional ventilation map to produce a 1D airflow having specified boundary conditions at each point. The boundary conditions at the start of the 1D airway tree were then imposed on the distal ends of the central airway tree. Uniform velocity and pressure boundary conditions were also implemented on this airway tree model simulation and compared to the patient-specific boundary model simulation. Large eddy simulation (LES) was utilized to capture transitional and turbulent flow in the central airway. The results showed that the distributions for pressure and velocity are more heterogenous for the patient-specific model than for the uniform velocity and uniform pressure boundary condition models (Figure 5). The uniform pressure boundary condition yielded a uniform pressure drop in all five lobes, the uniform velocity boundary condition yielded a greater pressure drop in the right middle lobe than the others, and the patient-specific boundary condition yielded a greater pressure drop in both the left lower lobe and the right lower lobe. The distribution in the patient-specific model was consistent with what is expected from the airflow distribution in heterogenous lung anatomy [2, 16].

The patient-specific boundary condition at the distal end of the airway tree is a flow rate boundary condition. From the CT intensity at the distal airways for the images acquired, the air fraction at each voxel can be calculated as [62, 63]:

(15) $$\beta_{\text{air}}\left(x\right)=\frac{{\text{HU}}_{\text{tissue}}-I\left(x\right)}{{\text{HU}}_{\text{tissue}}-{\text{HU}}_{\text{air}}}$$

where $\beta_{\text{air}}\left(x\right)$ and $I\left(x\right)$ represent the air fraction and image intensity at a given voxel location x. ${\text{HU}}_{\text{tissue}}$ (parenchyma and blood) and ${\text{HU}}_{\text{air}}$ represent the Hounsfield units for tissue (+65) and air (−1000), respectively. The value of the air fraction at each distal voxel is multiplied by the reference volume of a CT image voxel to get the volumes of air at any two-time points during inflation. The difference in volumes over the difference in time gives the air flow rate at each distal airway tree ending [16].

Lin and coworkers [5, 16], as well as Tawhai and coworkers [5, 64, 65] proposed a more detailed computational framework. In addition to performing simulations using the gray level values from the image scans for the patient-specific boundary conditions, a free-form deformation and a soft tissue deformation technique can be utilized to attain subject specific boundary conditions. For the free-form deformation, images at two time points (*t*_1_ and *t*_2_) during inflation are acquired and the image at *t*_1_ is embedded with a finite element mesh. The 3D image at *t*_1_ is then transformed into the *t*_2_ image and the change (transformation) in the volume is assumed to be equivalent to airflow. Hence, the rate of change in volume from *t*_1_ to *t*_2_ is utilized as the flow boundary condition. The free-form deformation technique assumes that although there is a change in lung tissue form and structure, there exists no net change in tissue volume hence the volume change seen during inflation is attributed to air. The soft tissue deformation technique is used in calculating local expanding boundary pressure through finite deformation elasticity values of stress and strain of the lung parenchyma. The modeling framework was formulated to link equations for large deformation of the lung tissue to equations for airway flow and pressure at different regions in the airway tree.

Furthermore, the breathing cycle (inspiration and expiration) either during spontaneous breathing or assisted breathing via a mechanical ventilator not only involves airflow through the airway tree but also airway tissue deformation (contraction or expansion) at different regions. Hence, CFD simulations closer to physiological reality would include some form of fluid-structure interaction (FSI) [66, 67] which is lacking in some of these studies. Xia et al. developed and deployed an FSI technique that utilizes a finite volume-based and element-based structural dynamics method with a dynamic level-set meshing algorithm [68]. This FSI technique is triple based with the fluid domain, the airway structure domain, and a moving mesh. Navier stokes equation is first solved for the fluid domain and the results for external forces are applied to the airway structure domain to solve for tissue deformation. The deformed tissue structure provides new boundary conditions at the fluid-structure interface and prior boundary conditions for the fluid domain are updated. The process is iterative until convergence is attained for the entire simulation.

## 5. Ventilation and Perfusion

Thus far, the focus of this report has been on how airflow distribution can be modeled in the airway tree, since airflow distribution defines ventilation distribution. However, pulmonary pathophysiology also affects the distribution of perfusion, resulting in potential mismatch of ventilation-to-perfusion. Note that such mismatch also exists when perfusion is perturbed. Alveolar ventilation ${\dot{V}}_{A}$ is the movement of air to and from the blood-gas barrier, while pulmonary perfusion $\dot{Q}$ is blood flow in pulmonary blood vessels. For effective oxygenation of a living organism, both ${\dot{V}}_{A}$ and $\dot{Q}$ must be appropriately matched. Regional ventilation with poor perfusion results in physiologic dead space, which leads to wasted ventilation, while poor regional ventilation with normal perfusion results in shunting. Both conditions impair the uptake and elimination of gases by the lung [34, 43].

CT imaging plays a tremendous role in elucidating lung perfusion and its distribution throughout the vascular tree. Maps of ventilation and perfusion in the lungs can be studied concurrently, to analyze ${\dot{V}}_{A}/\dot{Q}$ relationships at different lung regions, volumes, and times during inspiration. It is assumed that for regions in the lung with little or no ventilation, the constriction of blood vessels in poorly ventilated regions and redistribution of blood to better-ventilated regions of the lung (i.e., hypoxic pulmonary vasoconstriction) maintains appropriate ${\dot{V}}_{A}/\dot{Q}$ matching. But this is not always the case. Si-Mohamed et al. [69, 70] noted that lobes with early pulmonary diseases were hyperperfused, while lobes with late pulmonary diseases were hypoperfused. Grillet et al. [70, 71] reported hyperperfusion in both early and late-stage pulmonary diseases, and Lang et al. [70, 72] reported a mixture of both hyperperfusion and hypoperfusion. These contradictory findings make it extremely important for pulmonary ventilation-perfusion relationships to be studied on a patient-specific basis, and for computational models to be developed to enhance knowledge in this field.

## 6. CFD Validation

Accuracy and reliability of computational simulations for gas and blood flow in the lungs rely on the robustness of the numerical techniques for solving the equations of motion and continuity, as well as the appropriateness of the imposed boundary conditions. Validation of such *in silico* simulations becomes crucial for researchers to ensure the credibility of their computational designs and assumptions. Among such validation techniques, *in vitro*, *in vivo*, and *ex vivo* experiments are often used for assessing and validating the fidelity of computational models used to simulate airflow mechanics within the lungs. By conducting experiments on physical lung models (*in vitro*), utilizing lung tissue samples (*ex vivo*), or functional imaging (*in vivo*), researchers can quantitatively evaluate the similarity between experimental measurements and computational predictions, thereby establishing a validation framework for computational simulations.

Li et al. [73] validated computationally simulated human nasal airflow under breathing flow rates of 180, 560, and 1100 mL s^−1^ with an *in vitro* experimental setup utilizing the right nasal cavity of a healthy male adult obtained from [74]. For all simulations, the inlet and outlet boundary conditions were set to match the experimental flow conditions. The numerical results of velocity profiles and turbulence intensities were obtained using the laminar model, RANS (k-ε, standard k-ω, SST k-ω, and RSM), LES, and DNS models. For both laminar and all RAN models, second-order spatial discretization schemes were used to solve pressure and momentum, and SIMPLEC was used for pressure-velocity coupling. For the LES model, the central difference scheme was used to solve pressure and momentum, and PISO was used for pressure-velocity coupling. For the DNS model, an in-house immersed-boundary-method-based solver was utilized to solve the Navier-Stokes equations, and an Adams-Bashforth and implicit Crank-Nicolson scheme were utilized to discretize the convective and diffusive terms respectively. Although there was irregularity in the flow field, the laminar model achieved good agreement with experimental results under restful breathing condition (assumed at 180 mL s^−1^) and performed better than the RANS models. As the breathing flow rate increased and turbulence was attained, the RANS models achieved more accurate predictions, but performed worse than LES and DNS. Both the LES and DNS models provided accurate predictions of the nasal airflow under all flow conditions, but were computationally expensive.

**Figure 6.**
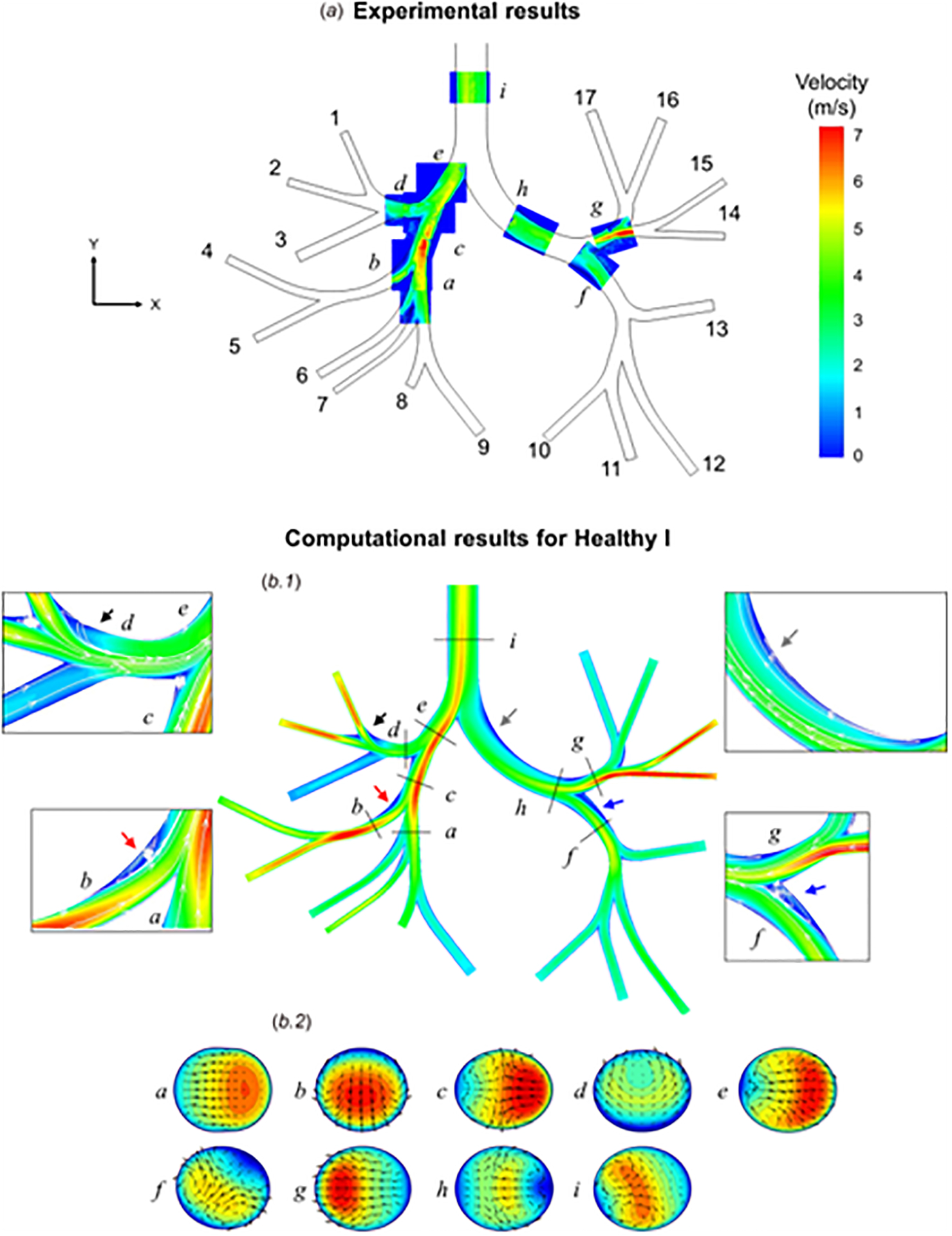
Flow velocity contours measured experimentally by PIV (a) and computed from a CFD model (b1 and b2), using boundary conditions from the *in vitro* study. (a) The in-plane velocity was evaluated in the coronal plane at the center of the geometry (z = 0). Velocities at the outlets (labeled numerically) and segments (labeled alphabetically) are shown. (b1) Velocity magnitudes in coronal section were derived from the CFD model using a healthy boundary condition. Flow streamlines are displayed with arrows highlighting areas of flow separation and recirculation. (b2) Axial velocity magnitudes and directions (vectors) are shown across branches a–i. Color bar indicates the velocity spectrum, with red corresponding to maximum velocity and blue corresponding to minimum velocity. From reference [75], with permission.

Sul et al. [75] utilized an extended model (idealized) of the lung airway structure to assess and validate airflow sensitivity to healthy and diseased lung conditions. The airway model included the upper airway, the central airway, and the distal airway up to the sixth generation. The upper airway was reconstructed from a CT image scan while the central and distal airways were based on the Horsfield model [12, 75, 76]. The same geometric model was utilized for *in vivo* (3D printed) and *in silico* studies. Airflow was simulated in both the *in vivo* and *in silico* studies under steady exhalation conditions with the total airflow rate at 770 m s^−1^, corresponding to flow rate under moderate activity. RANS model was utilized to solve for turbulence in the flow field for the *in silico* simulation. Particle Image Velocimetry (PIV) was implemented to capture flow fields and flow measurements were obtained from the PIV images. The velocity contours obtained from PIV and computational simulation measurement were shown to be similar with slight regional variations in velocity magnitudes (Figure 6). The PIV measure and computed velocity contours were evaluated at regions *a-i*. Regions *e* and *i* did not agree, although the velocity profile in both conditions for region *e* was asymmetric. The velocity magnitude obtained for region *e* from the computational simulation was 19% higher than that in the *in vitro* experiment. In region *i*, the velocity profile was positively skewed for the *in vitro* experiment whereas it was centralized for the *in silico* study, although both studies show that the contour was more influenced by the flow from the right lungs (regions *a, b, c, d*, and *e*). Nonetheless, all other regions analyzed had similar velocity contours and magnitudes for the *in vitro* and *in silico* studies.

Functional CT imaging techniques can also be used to validate computational fluid dynamic simulations of lung airflow more accurately [77–80]. Xenon-enhanced CT imaging is a specialized CT imaging technique that utilizes xenon (Xe), a dense noble gas, as a contrast agent during imaging [34]. By introducing xenon into the airway during imaging, this technique enhances the CT intensity voxel values, enabling the tracking of airflow. Dynamic contrast enhancement occurs during the Xe wash-in process, allowing for improved visualization and analysis of airflow dynamics [78]. While there have been no direct studies comparing xenon-enhanced CT imaging and computational simulations of airflow in the lungs, both techniques have independently been used to evaluate gas transport in the lungs. With the advancement in high-speed computing in respiratory mechanics and access to CT scanners, future studies may be carried out to explore the integration of xenon-enhanced CT imaging and computational simulations of airflow in the lungs.

MRI with hyperpolarized gases is another functional imaging technique that can be used to validate the computational fluid dynamic simulations of the lung. While MRI has limited ability to depict lung anatomical details because of its long acquisition time and the lungs’ low tissue and low proton density, it can still be optimized and serve as an alternative for CFD validation. Similarly to xenon-enhanced CT imaging, hyperpolarized (i.e., helium or xenon gas) MRI provides a way to visualize the distribution of airflow in the lungs without ionizing radiation [81]. The validation of CFD using hyperpolarized MRI for CFD has also been explored [79, 80].

These validation techniques play a pivotal role in guiding researchers in the development of accurate and reliable computational models, enabling deeper insights into complex airflow phenomena in the lungs.

## 7. Conclusions

In this review, we noted several limitations of computational fluid simulation: (1) the geometric models used are not often representative of the human lung, (2) constant inlet values are often utilized instead of oscillatory values, (3) outlet conditions are often constant and set to zero, and (4) geometric meshes are usually assumed to be stationary during the breathing cycle rather than dynamic, neglecting fluid–structure interaction. These simplifications are often utilized because of the complexity and computational expense of simulating representative flow problems. Despite these challenges, computational methods for simulating gas flows in the mammalian airway tree are useful for assessing the effect of various structural derangements on pulmonary function. While much remains to be done in developing pipelines for effective and efficient geometric models that are valid for simulation and representative of airway anatomy, the current models and simulations have shown promising agreement with the respective validation techniques. These findings imply that computational simulations can provide meaningful insights into airflow mechanics and contribute to our understanding of the respiratory system in health and disease. Although there have been ongoing improvements to the limitations discussed above, continued refinements to simulation techniques will yield more accurate and reliable results. Future simulations should utilize anatomic geometric models, unsteady inlet boundary values, non-zero and unsteady outlet boundary values, dynamic meshes that account for fluid–structure interactions, and high-speed computing resources to ensure accuracy while saving computational expense.

## Glossary

*d*: Branch diameter
*f*: Flow-dividing ratio
HU_tissue_: Tissue Hounsfield Unit
HU_air_: Air Hounsfield Unit
*I*: Image intensity of a given voxel
*I*_*b*_: Mean intensity value of body voxels
*I*_n_: Mean intensity value of non-body voxels
k-ε: k-epsilon turbulence model
k-ω: k-omega turbulence model
*L*_*e*_: Entrance length
*n*: Diameter exponent
$\dot{Q}$: Perfusion
*r*: Branch radius
Re: Reynolds Number
SST: Shear Stress Transport
*T*_0_: Initial gray-level threshold value
*T*_*i*_: Gray-level Threshold value at the *i*^th^ iteration
$\overset{-}{v}$: Mean velocity
$\dot{V}$: Volumetric flow rate
${\dot{V}}_{A}$: Alveolar ventilation
*x*: Voxel location in 3D image
α: Womersley Number
β_air_: Air fraction within a voxel
Δ: Airway recursion index
Θ: Airway bifurcation angle between parent and daughter branches
μ: Viscosity
ρ: Density
ω: Angular frequency
